# How digital transformation can accelerate data use in health systems

**DOI:** 10.3389/fpubh.2023.1106548

**Published:** 2023-03-15

**Authors:** Laurie Werner, Chilunga Puta, Taonga Chilalika, Sara Walker Hyde, Hannah Cooper, Hallie Goertz, Maya Rivera Hildebrand, Christina Bernadotte, Veronica Kapnick

**Affiliations:** ^1^PATH, Seattle, WA, United States; ^2^Cooper/Smith, Austin, TX, United States; ^3^Great Impacts Consulting, Seattle, WA, United States

**Keywords:** digital health, health data, health information system, Tanzania, South Africa, Malawi, Ethiopia, Burkina Faso

## Abstract

**Introduction:**

We are in an era of rapid technological advance and digitalization. Countries around the world want to leverage technology to improve health outcomes by accelerating data use and increasing evidence-based decision-making to inform action in the health sector. Yet, there is no “one size fits all” approach to achieving this. To understand more, PATH and Cooper/Smith conducted a study documenting and analyzing the experiences of five African countries (Burkina Faso, Ethiopia, Malawi, South Africa, and Tanzania) that are on this digitalization journey. The goal was to examine their different approaches and develop a holistic model of digital transformation for data use that identifies what the essential components for digitalization success are and how they interact with each other.

**Methods:**

Our research had two phases: first, we analyzed documentation from the five countries to identify core components and enabling factors for successful digital transformation, as well as barriers encountered; and second, we held interviews with key informants and focus groups within the countries to fill gaps and validate findings.

**Findings:**

Our findings show that the core components of digital transformation success are highly interrelated. We found that the more successful digitalization efforts address issues that cut across components—such as stakeholder engagement, health workforce capacity, and governance structures—and consider more than just systems and tools. Specifically, we found two critical components of digital transformation that have not been addressed in previous models like the eHealth strategy building blocks developed by the World Health Organization and the International Telecommunication Union: (a) cultivating a culture of data use throughout the health sector and (b) managing the process of system-wide behavior change required to move from manual or paper-based to digital systems.

**Conclusion:**

The resulting model is based on the study's findings and is intended to inform low- and middle-income (LMIC) country governments, global policymakers (such as WHO), implementers, and funders. It provides specific, concrete, evidence-based strategies these key stakeholders can implement to improve digital transformation for data use in health systems, planning, and service delivery.

## Introduction

The use of reliable, robust data plays an integral role in public health planning, resource allocation, and policymaking (1). To improve the use of data in the health sector, many low- and middle income (LMIC) countries work with partner organizations to replace their existing paper-based health data collection systems with digital ones, such as logistics management information systems, electronic medical records, or more simple electronic patient registers. Such systems can reduce errors and redundancies and increase the sharing of data across health facilities and providers to facilitate greater access to data by those who need it to make decisions and take actions. This process of *digital transformation for data use* involves moving these paper-based processes for data collection, management, reporting, and analysis to digital tools and formats. It encompasses every aspect of health information systems (HIS), including human resources, business processes, technology, and workplace capabilities (2).

However, digital health systems in many resource-constrained countries remain largely disconnected from one another, underdeveloped, and underused (3). For health data to improve service delivery and outcomes, international donors and country governments must invest in digital infrastructure and country capacity to manage the nation's data collection and processing independently (1). Introducing new tools and systems must be accompanied by a commitment to developing a culture of data use that promotes the systematic gathering, analysis, reporting, and assessment of high-quality data to guide health system decisions and actions (4, 5). The use of data can lead to improvements in delivery of health services. One study in Mozambique, Rwanda, and Zambia showed an increase from <10% to >80% adherence to basic clinical protocols with the introduction of data use plans and skills for health workers (6). To achieve such use of data and build a strong culture of data use, in addition to the introduction of connected tools and systems, several factors must be addressed with the data users themselves, including strengthening their skills and knowledge, as well as their understanding of the value of the data to their work (7).

**Figure 1:**
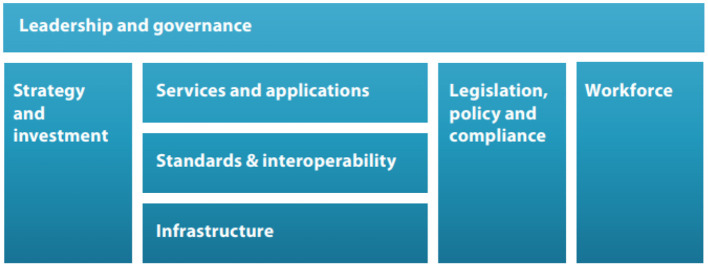
WHO-ITU eHealth strategy building blocks. Source: World Health Organization (WHO), International Telecommunication Union (ITU) (8).

In 2012, the World Health Organization (WHO) and the International Telecommunication Union (ITU) identified seven fundamental eHealth strategy building blocks for supporting the growth and maintenance of countries' digital health environments (8) (see Figure 1). Other frameworks and models also have been developed related to digital health and the use of health data. These include the Performance of Routine Information System Management (PRISM) series from MEASURE Evaluation (9), which looks at technical, organizational, and behavioral determinants of routine HIS; the Immunization Data: Evidence for Action (IDEA) theory of change (5), which focuses on the factors and interventions that influence data use; and the Data Use Partnership theory of change (10), which examines factors that accelerate the use of data for improved health system performance. Each of these models has played a critical role in informing our understanding of digital health systems and their users, and many models have been used to inform financing mechanisms, normative guidance, and programming approaches. However, the picture they articulated of what is needed to digitalize a health system was not complete (11) and few of these models were built on direct country experiences (although they have been used since in many countries). Therefore, the innovation and lessons that emerged from countries' digital transformation experiences have not always been included or reflected in the approaches of international partners and policymakers working in the digital health space.

Using these existing models as a starting point, PATH and Cooper/Smith, with support from the Bill & Melinda Gates Foundation, worked to document and analyze countries' experiences, studying the practices in five African countries that are digitalizing their health systems: Burkina Faso, Ethiopia, Malawi, South Africa, and Tanzania. The goal of this study was to formulate a holistic model of digital transformation to accelerate data use from which other governments and key stakeholders could learn.

The study aimed to explore the following research questions:

- What are the best practices that contribute to having countries increase and accelerate data use?
- What are the challenges encountered with planned activities, and how did countries address them?
- How did the digital transformation work link directly to or influence each country's broader strategy, plans, or priorities?
- Did any common factors either contribute to or deter digital transformation for data use across the five countries?
- What lessons, practices, and innovations can be broadly shared with countries undertaking digital transformation work?

Using qualitative analysis of documentation and interviews with key informants, the study identified and validated critical enabling factors, tools, and approaches. The results of the study demonstrate the value in taking a holistic approach to digital transformation, considering the health system as a whole. Country governments, and the funders and implementers they work with, need to be aware of the interconnected components of digital health transformation for data use—and the country's overall vision—and strive to align with and support work that is planned or already underway.

By sharing these findings with countries and the global community, we intend to accomplish the following:

- Inform country governments of the most critical considerations for digital transformation work.
- Advise implementers on how best to support countries in digital transformation efforts.
- Inform guidance and policies developed by policymakers relating to digital transformation and data use.
- Guide funders in making investments that align with country strategies and visions and coordinate with other funding and digital transformation efforts.

## Methodology

### Research design

To achieve the objective of this study—identify the key components of a model of digital transformation to accelerate data use based on experiences in the five focal countries—we used the following research process:

1. Conducted a review of existing models and frameworks related to digital health and the use of health data. The review allowed us to identify common themes across existing models and frameworks related to data use, digital health, and HIS.
2. From the existing models and frameworks review, we developed a hypothesized framework incorporating the core components of digital transformation for data use, building upon the existing work to date, in collaboration with partners from the five countries.^1^
3. Mapped the experiences and lessons learned in the five focal countries to the hypothesized framework (see Table 1) through a document review and identified gaps in evidence to inform primary data collection.
4. Interviewed key informants to further build the evidence base and understand the contributing factors to achievements in digital transformation for data use.
5. Analyzed the evidence to identify themes within each country and then across all five countries to inform a model for digital transformation.
6. Conducted a cross-country conversation to further understand and validate findings. This virtual conversation included representatives from the five countries, to review the key themes that emerged across the countries, validate those themes, and further elaborate on the key themes as a group. The discussion provided inputs that were incorporated into the final analyses across the five countries and validated the themes that emerged from the two phases of research.

**Table 1:** Framework for digital transformation for data use.

**WHO/ITU eHealth building block or other core component**	**Subcomponents**
Leadership and governance	• Committed leadership and champions • Multisector engagement • Governance structures
Legislation, policy, and compliance	• Priority policy areas • Security standards, privacy/confidentiality in data sharing, and related legal frameworks • Compliance and enforcement
Strategy and investment^a^	• Strategy • Investment
Standards and interoperability	• Standards and interoperability • Enterprise architecture
Services and applications	• Applications • Technical support for systems and users
Workforce	• Data and digital literacy and capacity • Motivation and incentives
Infrastructure	• Physical infrastructure and system maintenance (servers, computers, tablets, backup paper supplies, etc.) • Energy/electricity • Connectivity
Data use ecosystem^b^	• Data collection, management, analysis, and dissemination • Feedback on data and data use • Data quality • Accessibility to relevant data users
Change management^b^	• Change management

^a^Not one of the original WHO-ITU eHealth strategy building blocks. ^b^Divided into two components in the final model that emerged as a results of the work described in this article.

We developed the hypothesized framework base on the review of existing models and frameworks, specifically the WHO-ITU eHealth strategy building blocks (8), as well as other key resources and models, including the Tanzania Data Use Partnership theory of change (10), the PRISM framework (9), the IDEA report (5), and several change management models (12–14). In all, we reviewed and included 36 documents in developing this framework. Table 1 lists the components of the hypothesized framework that we developed and subcomponents we identified for each.

### Phase 1: Secondary data collection

The initial research phase in early 2021 consisted of secondary data collection, during which we gathered evidence from the key country stakeholders, documented lessons learned, and identified best practices from existing documentation of current and past digital transformation investments in the five focal countries. We used this evidence to identify gaps in information that would inform a more targeted Phase 2 primary data collection.

During Phase 1, we compiled a total of 72 documents from across the five countries. We coded and analyzed them in ATLAS.ti software using a coding structure modeled on the core components of the hypothesized framework. This allowed for a consistent review across documents and provided a systematic way to capture and categorize information from all five countries. The documents also were tagged by country and type of document and included grant proposals, donor reports, investment road maps, digital health strategies, HIS policies and guidelines, communication products, and evaluation reports.

To create a foundational picture of what had occurred, identify the initial lessons learned and best practices, and inform focus areas for further investigation through the Phase 2 primary data collection, we also coded for the following crosscutting themes during the document review:

- Broader country-level national strategies and priorities and how they were linked with digitalization efforts.
- Plans and scope for investment (goals, milestones, expected outputs and outcomes, level of investment, etc.).
- Timeline of major project activities, as well as events that may have affected implementation (shifts in leadership, local epidemics or pandemics, etc.).
- Achievements, both measured and perceived (narrative, qualitative, or quantitative).
- Key changes or challenges with plans and activities and their causes.

We then synthesized and analyzed Phase 1 data from the document review to identify any gaps in understanding a specific country's experience regarding the core components of the framework. We validated these gaps with key stakeholders as areas for further exploration during Phase 2 through interviews and validation workshops.

### Phase 2: Primary data collection

To fill the information gaps identified during Phase 1, we conducted key informant interviews and focus group discussions addressing the following topics and overall lessons learned:

- Validation of digital transformation activities in the country identified in the secondary data collection.
- Accomplishments and achievements and what respondents believed contributed to them.
- Main challenges or changes experienced, what contributed to them, and what respondents did to address or adapt to them.

We developed a comprehensive interview guide for these discussions that was then tailored for each country and pre-tested the questions before use. Questions focused on key achievements, where they have seen change and what the contributing factors or enablers for those changes were, and what are the challenges they face with their digital transformation and data use efforts.

We held interviews between May and July 2021 in compliance with the guidelines for conducting research activities during the COVID-19 pandemic. The number and types of interviewees differed by country depending on the specific information needed (see Table 2 below), respondents' experiences within the country, and respondents' relationships with the digital transformation work. Interview duration ranged from 1–2 h, depending on the stakeholder and language. Respondents participated in the study on a voluntary basis and comprised adults across genders and age groups. We conducted the interviews with participants' verbal consent while ensuring anonymity in analysis, reporting, and dissemination.

**Table 2:** Number of documents and interviews analyzed.

**Country**	**Documents**	**Interviews**
Burkina Faso	8	3
Ethiopia	21	10
Malawi	18	7
South Africa	8	7
Tanzania	17	6
Total	72	33

Examples of interviewees included:

- Ministry of Health (MOH) officials.
- National data management officials.
- Program managers.
- Technology and innovation officials.
- Workers in health facilities.
- Implementing partners of digital transformation projects.
- Other partners or organizations that contributed to digital transformation activities.
- Donors.

Interviews conducted in English were recorded and uploaded to an online transcription platform (TEMI.com). Interviews conducted in languages other than English (such as Amharic, Swahili, or French) were recorded and then translated to English, and interviewers and note takers documented relevant information for each question in English. We tagged documents as either transcripts or notes for coding using ATLAS.ti, retaining codes from Phase 1 (with minimal updates), and stripped identifiable information from the transcripts to ensure confidentiality.

We ensured quality control of data through training of data collectors, training on the codebook, pretesting data collection tools, and reviewing data completeness as data were entered into data collection tools. In addition, coders for Phase 1 and Phase 2 remained the same, and two people coded the same documents for the first batch to ensure intercoder reliability; this was followed by weekly check-ins across the coding team to confirm consistency in coding. Training included consent practices and COVID-19 safety precautions.

### Data analysis

We analyzed data from both Phase 1 and Phase 2 in two stages: within each country and across all countries. The numbers of documents and interviews coded and analyzed for each country are shown in Table 2 above.

We examined frequencies and relative frequencies of coded themes first within countries and then across countries, document types, and respondent types (for key informant interviews) to validate our interpretations and guide reexamination of data when discrepant patterns suggested unexplained anomalies. We shared each country's final analysis with its respective stakeholders to validate the findings and gather any final clarification and input. We then identified emerging themes, as well as differences and similarities, among the countries. We conducted a virtual cross-country discussion with 22 attendees from the five countries to further identify commonalities, contributing factors to success, and challenges, as well as to validate country experiences. Finally, we conducted virtual workshops convening country officials, policymakers, donors, and implementers to discuss and validate the overall findings. The outputs of the data analysis, cross-country conversations, and workshops were synthesized into a comprehensive model of the critical factors for implementing digital transformation to accelerate data use (15).

## Findings

**Figure 2:**
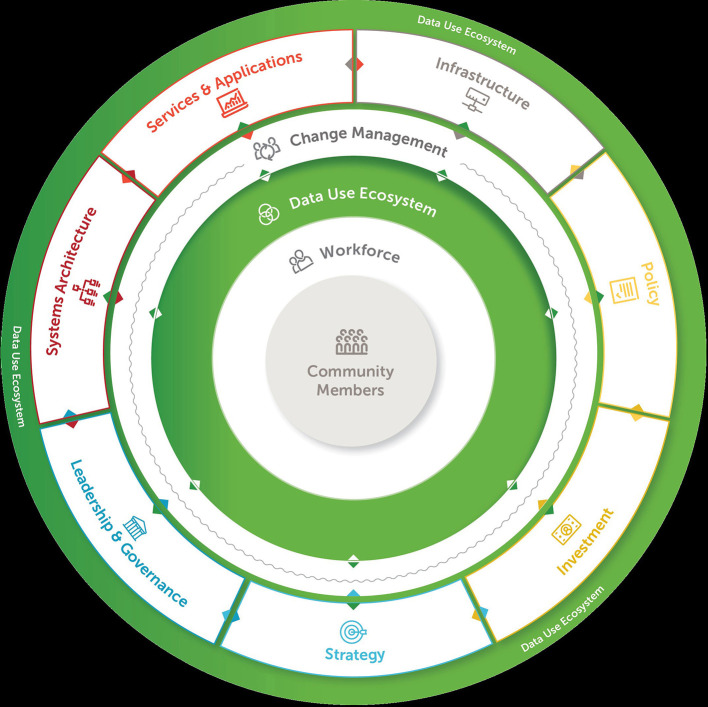
The data use acceleration and learning (DUAL) model.

Using evidence from countries' direct experiences, we identified ten key components of digital transformation to accelerate and improve data use in the health sector (see Figure 2). Our analysis shows that, overall, the core components of successful digital transformation proved to be relevant in the five countries' experiences, as well as highly relational in the process to accelerate data use. When components such as leadership and governance, strategy, and investment work in alignment, they act as enablers for achieving digital transformation goals. Therefore, successful efforts to advance data use depend not just on putting tools and systems in place within the health sector but also on improving areas that affect, and are affected by, these technologies, such as policies, infrastructure, and capacity-building for the health workforce.

We identified key enabling factors for each core component based on evidence of what worked and what presented barriers to success in the five focal countries we studied. Because the core components are highly interrelated, many important enabling factors cut across multiple components. Specifically, the following key enabling factors were seen in multiple of the core components of successful digital transformation for data use:

- **Engage stakeholders and develop digital health champions, including coordinating and aligning stakeholders**. In many cases, stakeholder engagement and coordination relied on working with existing technical working groups or forming new ones to solve issues and implement solutions. Stakeholder engagement also included holding forums and creating communities of practice with key partners, obtaining leadership buy-in, and fostering the collaboration necessary to develop and implement project activities across different partners. Respondents in several countries noted that having the right people at the table throughout the process of engaging stakeholders, particularly those who were technically savvy and well qualified, was an important facilitator to success. As one interviewee noted, the “right people” are not always located within the country's MOH, and local government authorities, departments, and ministries outside the health sector should also be included.

Champions within government who could provide a conduit to other leaders and rally stakeholders to advance project objectives were seen as necessary allies in digital transformation activities. In Ethiopia, implementation teams identified government champions to raise awareness of such activities. For example, the state minister's office led the development of new standards, governance, and enforcement mechanisms for Ethiopia's digital health systems, helping to build support at the highest levels of leadership. A key informant in Ethiopia noted that having invested leadership facilitated the creation of a culture of data use: “The more the leader is engaged, the more the leader is proactive. The more the leader uses data, [the more] likely [he or she is to act in] supporting … others … to use data.”

- **Establish clear, strong governance structures appropriate to the country's context and needs**. A noted challenge to implementing new tools and applications was the lack of a governing body to oversee the review, approval, and coordination of different projects. In Tanzania, the implementing partners and the Ministry of Health, Community Development, Gender, Elderly, and Children established a project governance team, which included funder representation, to manage the work conducted across multiple ministries and agencies. In addition, a National Digital Health Steering Committee, chaired by the permanent secretary of the MOH, was formed to oversee the national digital health strategy. Ethiopia and Malawi both adopted an embedded approach that placed project implementation staff directly within government operations. This approach helped to build champions within the MOHs, determine processes for decision-making, and coordinate digital projects and investments.
- **Apply user-centered design approaches in identifying and implementing digital tools and systems** (16). These approaches varied across countries but ranged from conducting data user studies (in Malawi and Burkina Faso) to holding focus groups with health workers to gain insight into user needs (South Africa). In South Africa, consortium meetings, stewarded by the government, were held to discuss the planning and implementation of digital health solutions. These meetings allowed technical staff and government officials to discuss new technologies with peers who had strong on-the-ground and domain knowledge. Similarly, Tanzania used an “agile methodology” (17) in their strategic planning efforts, working directly with users to develop systems and tools, which was viewed as critical for successful implementation.
- **Improve training of the health workforce to build workforce capacity**. Most of the five countries studied included some form of capacity-building in their activities to improve use of digital tools and data by health workers. For example, the government and partners launched a data use campaign in Malawi to increase health workers' access to data and their knowledge of data systems. In Ethiopia, the government and partners established a learning center where health workers could receive technical and professional support. Dashboards, which were used in Malawi and Ethiopia, were also noted as a viable strategy to improve data access and use by helping to process data into useful information for health workers. Both Ethiopia and Tanzania worked with universities to develop and standardize training curricula for preservice and in-service training in HIS and data use. In Tanzania, the government also updated its e-learning platform for in-service health workers in remote areas.
- **Ensure data are collected, shared, and monitored across systems**. This was primarily done by establishing data standards, inventorying systems in use, and increasing the interoperability of different systems and tools. For example, in Tanzania, a health enterprise architecture (18) was developed by the government to serve as a conceptual blueprint for the structure and operation of the country's digital health systems (19). The MOHs in Ethiopia and Burkina Faso inventoried digital health applications in use to help improve coordination among projects and avoid duplication of effort. Malawi's implementation team worked with the MOH to align national data standards and district-level requirements, which enabled a more efficient systems architecture.
- **Align funding**. When donor coordination under a common plan or vision is lacking, investments may not align with achieving the country's digital health plans, as one key respondent in South Africa noted. To help align funding and digital health priorities in Tanzania, the government created a digital health investment road map that recommended areas for investment, as well as financing and cost guidelines. Another strategy for coordinating different investments was to include funders and donors in technical working groups. All five countries included donors in their stakeholder engagement to varying degrees.

## Discussion

Our model for digital transformation builds on the WHO-ITU eHealth strategy building blocks (8) and other existing frameworks but introduces two new critical factors for advancing data use: the data use ecosystem and change management strategies that were not included in the original set of building blocks. The data use ecosystem comprises all the activities that improve access to and use of data, including data collection, quality, demand, and analysis (5, 11). Our research has found that cultivating a culture of using data for evidence-based decision-making and action throughout the health sector is foundational to success. Our model also acknowledges the essential role of employing proven strategies to introduce new technologies, systems, and processes to the health workforce to ensure a smooth transition and widespread adoption.

Further, our model emphasizes that all of the core components enable and support one another in advancing digital transformation to improve data use, with the critical focus on the community and people served by the health system at the center. As shown in Figure 2 above, digital transformation is not a simple, step-by-step process. Rather, it is complex and iterative, where many components rely upon or are affected and supported by other components. Our model can be used by country governments to identify gaps in their plans and strategies and take steps to address them. Countries can also use the model to target investments toward the most critical areas and better align their digital transformation work across stakeholders and partners, thus increasing chances for sustained impact.

Specifically, we recommend the following actions be taken to improve digital transformation efforts in countries:

- **Engage stakeholders and improve leadership**:
  ° Country governments can form technical working groups across sectors.
  ° They can cultivate digital health champions at all levels of the health system.

- **Cultivate a culture of data use throughout the health system**:
  ° Country governments and implementers can build the capacity of all health workers through training, professional development, mentoring, and other proven practices.
  ° They can motivate and empower health workers to use and act on data, rather than just serve as data collectors.

- **Strengthen governance structures**:
  ° Country governments can establish governance bodies to establish, manage, and enforce digital health policies, guidelines, and standards.
  ° Implementers can ensure that digital health activities are government driven and work within existing structures (such as technical working groups, steering committees, etc.).

- **Increase evidence-based planning:**
  ° Policymakers can use evidence to inform new and revised policies and guidance for digital health and data use.
  ° Funders can leverage data, assessments, and system evaluations to inform investment decisions.

- **Improve the design of health systems**:
  ° Country governments can advocate for building systems and tools that are responsive to infrastructure limitations and challenges.
  ° Implementers can apply user-centered design approaches and build multiuse systems rather than designing separate systems for each use case.
  ° Funders can invest in global goods, standards, and interoperable systems instead of standalone systems.

- **Align and sustain funding efforts**:
  ° Country governments can develop investment road maps and long-term funding streams.
  ° Funders can ensure investments are country led and align with countries' visions, priorities, and strategies.
  ° Funders can identify the true costs of digital and data infrastructure to help determine sustainable funding streams.

## Conclusion

Implementing digital health tools and information systems can improve health service delivery, but not if done without considering the broader goals, vision, and data ecosystem of the country's health system. Our model for digitally transforming health systems to advance data use, based on countries' actual experiences, emphasizes a holistic approach that considers not only tools, systems, and infrastructure but also strengthening governance, building the capacity of the health workforce to use these systems, systematically managing the transition to digital health systems, and cultivating a culture of data use throughout the health sector. We envision a future in which all stakeholders in digital health, both globally and nationally, work together to advance and accelerate digital transformation in alignment with current best practices and each country's health strategy and digital maturity to strengthen the health system and deliver high-quality health services.

## Limitations

Some limitations that affected our interpretation of the study's findings are worth noting. Each country we studied is at a different stage of development of its digital transformation and therefore has demonstrated greater or lesser achievements and progress compared with the other countries in the study. As such, some countries may be represented more than others in the findings, as their activities have been underway for longer periods. Another limitation is that, in some cases, key informants interviewed were individuals who either were part of the team overseeing digital transformation activities or worked closely in support of it. Therefore, there may be some bias in their experiences and insights. We have taken this into consideration by engaging in sense-making discussions with country stakeholders to ensure that specific views are not overrepresented.

## Data availability statement

The raw data supporting the conclusions of this article will be made available by the authors, without undue reservation.

## Author contributions

LW, CP, HC, and CB contributed to the conception and design of the study. SW, HC, MRH, CB, TC, and VK performed the analysis. LW wrote the initial draft. CB, TC, MRH, VK, and HG wrote and edited sections of the manuscript. All authors contributed to manuscript revision, read and approved the submitted version.

## Footnotes

^1^Partners included Cooper/Smith (Malawi and Burkina Faso), PATH (Tanzania), JSI Research and Training Institute (Ethiopia), and University of Cape Town (South Africa).
